# Histological Evaluation of Human Pulp Response to Direct Pulp Capping with MTA, CEM Cement, and Biodentine. 

**DOI:** 10.30476/DENTJODS.2019.81796.0

**Published:** 2020-09

**Authors:** Razieh Hoseinifar, Ali Eskandarizadeh, Masoud Parirokh, Molook Torabi, Fereshteh Safarian, Elina Rahmanian

**Affiliations:** 1 Oral and Dental Diseases Research Center, Dept. of Operative Dentistry, School of Dentistry, Kerman University of Medical Sciences, Kerman, Iran; 2 Dept. of Operative Dentistry, School of Dentistry, Kerman University of Medical Sciences, Kerman, Iran; 3 Dept. of Endodontic, School of Dentistry, Kerman University of Medical Sciences, Kerman, Iran; 4 Dept. of Pathology, Faculty of Dentistry, Kerman University of Medical Sciences, Kerman, Iran; 5 Dept. of Orthodontic, School of Dentistry, Kerman University of Medical Sciences, Kerman, Iran; 6 Dept. of Operative Dentistry, School of Dentistry, Zahedan University of Medical Sciences, Zahedan, Iran

**Keywords:** Dental pulp capping, Dental pulp, Inflammation

## Abstract

**Statement of the Problem:**

Direct pulp capping (DPC) is an established method in which the exposed pulp is coated with a suitable material to prevent further damage and to help its repair and healing. Different proposed materials may have different impact on pulp response during this treatment.

**Purpose:**

The purpose of this study was to compare the response of human dental pulp after DPC with calcium-enriched mixture (CEM), mineral trioxide aggregate (MTA) cement, and Biodentine.

**Materials and Method:**

In this clinical trial study, class V cavities were prepared on the buccal surfaces of 30 human premolar teeth, until the pulps were mechanically exposed. Then, teeth were randomly pulp capped with MTA, CEM cement and Biodentine, followed by resin modified glass ionomer filling. The fourth group was the control group (n= 10), in which the teeth were extracted without any prior intervention. Six weeks after the intervention, the teeth were extracted and prepared for histological evaluation in terms of the type and degree of pulp inflammation, dentin bridge formation and the presence of necrosis. Data were analyzed using Kruskal-Wallis and Mann Whitney U tests.

**Results:**

In all groups, necrosis was not observed and inflammation was chronic. The Biodentine group exhibited significantly more pulpal inflammation compared with the other groups (*p*= 0.001). There were no significant differences among CEM cement, MTA and Biodentine in terms of the dentine bridge formation. The thickness of the dentin bridge formed in the Biodentine group was significantly higher than MTA and control group (*p*= 0.035 and *p*= 0.011, respectively).

**Conclusion:**

Although the dentin bridge formation and the thickness of dentin bridge formed in the Biodentine group were higher than the other groups, pulp showed greater inflammation compared to CEM cement and MTA. The results of this study suggested that MTA and CEM cement performed better when employed as the direct pulp capping material.

## Introduction

It is generally accepted that maintaining the vitality of all or part of dental pulp is important, especially in traumatic teeth or those with an incomplete root [ 1 ]. Direct pulp capping (DPC) is a successful method for maintaining pulp vitality. During the DPC, the exposed pulp tissue is covered with a pulp-capping material to preserve its function and biological activities and to protect it from additional injury [ 2 , 3 ]. The success of DPC treatment is influenced by various factors such as patient's age, periodontal condition, root formation stage, pulp exposure dimensions and nature (traumatic, mechanical or caries), microbial contamination of exposed area, pulp status, and types and properties of pulp-capping materials as well as sealing ability of restorative materials [ 4 , 5 ]. Studies have shown that dental pulp healing after DPC is directly related to the properties of pulp-capping materials, including sealing ability, antimicrobial activity, biocompatibility, and the dentin bridge formation [ 6 , 7 ]. Over the past few years, various materials such as calcium hydroxide, biologically active molecules, Biodentine, calcium enriched mixture (CEM) and mineral trioxide aggregate (MTA) have been used for pulp capping [ 2 ]. MTA was introduced in 1993 by Torabinejad, which is the material of choice for conservative pulp therapy, apexification, and perforation repair [ 8 ]. MTA is composed of tricalcium phosphate, tricalcium aluminate, tricalcium oxide, silicate oxide, and several other oxides that are used in small amount in the composition of this substance [ 9 ]. A study has demonstrated excellent results for the MTA when used as a pulp capping material [ 10 ]. The desirable properties of MTA include excellent seal, high biocompatibility, low cytotoxicity, calcium ion release, and also its high alkalinity that provides its bactericidal properties. This material is not affected by contamination with blood or tissue fluids [ 4 , 10 , 11 ]. On the other hand, its unfavorable properties include delayed setting time, tooth staining over time, poor handling characteristics, and high costs [ 4 , 10 ]. 

CEM cement was first introduced as a root-end filling material. The main components of the cement powder are calcium oxide (CaO), sulfur trioxide (SO_3_), phosphorus pentoxide (P_2_O_5_) and silicon dioxide (SiO_2_) [ 12 , 13 ]. CEM cement is an effective antibacterial and antifungal agent and induces dentinogenesis, osteogenesis, and cementogenesis [ 6 ]. The CEM cement creates an environment with high pH value (about 11) and simultaneously releases high amounts of calcium and phosphorus ions, which facilitates the formation of hydroxyapatite [ 6 , 10 ]. The sealing ability and biocompatibility properties of CEM cement are similar to the MTA [ 10 ]. The CEM cement compared to the MTA has shorter setting time (less than an hour), similar pH, greater flow, less film thickness, good handling characteristics and no tooth discoloration [ 14 , 15 ]. 

Biodentine is also a new calcium silicate-based material, which has the same properties as the MTA, although it has resolved some of the problems of this material (setting time and staining) [ 16 , 17 ]. A study has shown that Biodentine has a positive effect on pulp cells and induces the formation of restorative dentin similar to MTA [ 16 ]. Biodentine has several good properties including good sealing ability, proper compressive strength, short setting time, and bioactivity. According to the manufacturer, this material has less setting time (around 12 minutes) than two other substances, which allows the placement of permanent restoration in shorter time [ 18 ]. 

Tabarsi *et al*. [ 14 ] examined the response of dental pulps in dogs to three pulp‐capping materials (MTA, CEM, and calcium hydroxide) and showed that there was no significant difference between CEM and MTA in terms of dentin bridge formation, pulp vitality, and lack of inflammation.

Nowicka *et al*. [ 16 ] evaluated the dentin bridge formation after direct capping with calcium hydroxide, MTA, Biodentine, and Single Bond Universal in human teeth and reported the maximum thickness of the dentin bridge was found when Biodentine was employed; they found no significant difference between the MTA and Biodentine in term of dentin bridge formation. 

Zarrabi *et al*. [ 15 ] compared the response of human dental pulps to MTA and CEM when used as pulp capping material and showed that CEM cement compared to the MTA provided a thicker dentin bridge, but their difference was not significant.

Despite the extensive research on pulp capping, previous studies have not yet used all of these materials (MTA, CEM cement, and Biodentine) in a clinical trial study and there is no complete comparison of these materials. Therefore, the aim of this clinical trial study was to compare the histological response of human dental pulp after direct pulp capping with three materials of MTA, CEM cement, and Biodentine.

## Materials and Method

The present randomized clinical trial study was conducted on 40 human first and second maxillary and mandibular premolar teeth without restoration, periodontal disease and caries, with healthy pulp from patients aged 14-25 years that were scheduled for extraction for orthodontic treatment. All clinical procedures were performed based on the Ethics Committee of Kerman University of Medical Sciences (Code of Ethics: IR.KMU.REC.1395.549 and IRCT code: IRCT2017091 728804N2).

Radiography was taken from each tooth at the baseline to rule out the possibility of any periapical lesions and proximal caries. The pulp vitality test was performed at the baseline by cold test and electric pulp tester. At the beginning, the teeth were divided into four groups, namely as (1) MTA group: pulp capping with MTA (Angelus-Brazil), (2) CEM cement group: pulp capping with CEM cement (Bionique Dent-Iran), (3) Biodentine group: pulp capping with Biodentine (Septodent-England), and finally (4) Control group.

 After local anesthesia with 2% lidocaine (Persocaine-E, Darou Pakhsh Co., Tehran, Iran), the teeth were isolated with a rubber dam, and then polished slowly with a rubber cup and prophylaxis paste, and disinfected by 0.2% chlorhexidine (Iran NAJO Pharmaceutical Co.). The class V cavities were prepared on the buccal surfaces of teeth using diamond straight fissure bur #57 (Diatech-Switzerland) in a high-speed handpiece (NSK, Tokyo, Japan) with copious water spray. The standard class V cavity was prepared with dimensions of 3mm of occlusogingival height, 5mm of mesio-distal width and axial wall depth at exposure limit of 0.5mm in diameter. The preparations were cut 1mm above the free gingiva. The pulp capping material was prepared according to the manufacturer's instructions, and then placed on the exposed pulp of the teeth according to their specified group. Then, the cavities were filled with resin modified glass ionomer (VOCO, Germany) in all groups. All procedures were performed by the same operator.

 In order to ensure the pulp vitality, the teeth were tested by the cold testing and electric pulp tester in 3 and 6 weeks after intervention. At the end of the 6th week (23,35), the teeth were extracted by premolar forceps without surgery and trauma. The extracted teeth were washed under sterilized water to remove blood and saliva. Then, the teeth were placed in 10% formalin (Merck, Germany) for 24 hours. After that, they were transferred into 15% nitric acid (Merck, Germany) for decalcification. The acid container was renewed daily, and the process continued to soften the tooth for the histological slide preparation process. The teeth were cut parallel to the longitudinal axis of the cavities, and placed in a special basket, and subsequently in the Tissue Processor (START-Japan). Then the paraffin blocks were prepared and 5 µm slices were cut from blocks by a microtome (Scilab-England). The sections were stained by hematoxylin and eosin (H &amp; E) staining method and were evaluated using an optical microscope (Olympus-Japan) with a magnification of 400X.The evaluation was performed by a blinded pathologist.

The criteria evaluated were the inflammatory response, necrosis, dentin bridge formation, dentin bridge thickness, and the type of inflammation. The grading scales for the inflammatory response were considered as (0): no inflammation (0-25 inflamed cells at the magnification of 400X), (1): mild inflammation (26-50 inflamed cells at the magnification of 400X), (2): moderate inflammation (51-75 inflamed cells at the magnification of 400X), and (3) severe inflammation (76-100 inflamed cells at the magnification of 400X).

The assessment for necrosis was graded as (0) for absence of necrosis, and (1) for presence of necrosis. Moreover, for dentin bridge formation the scale was (0) for absence of dentin bridge, and (1) for dentin bridge deposition beneath the exposure area; for dentin bridge thickness, the mean diameter of the dentin bridge formed in three regions in terms of microns was measured. The inflammation type was graded as (1) for chronic inflammation, and (2) for acute inflammation.

Data were analyzed by SPSS version 21 using Kruskal-Wallis and Mann Whitney U tests. The significance level was considered to be at p< 0.05.

## Results

**Figure 1:**
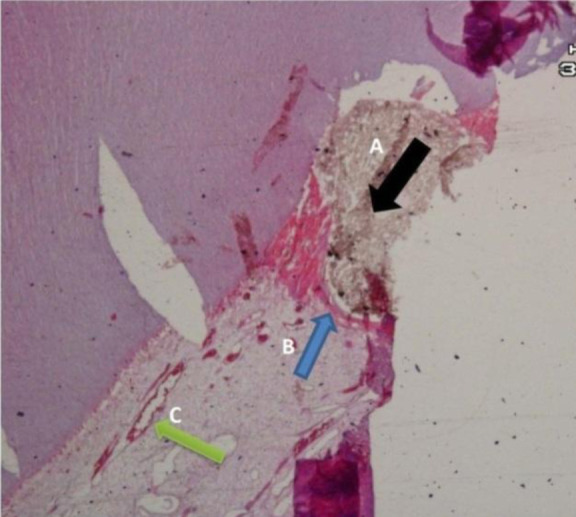
Pathologic image of a sample pulp capped with MTA: A (MTA material), B (calcified bridge), C (pulp hyperemia)

**Figure 2:**
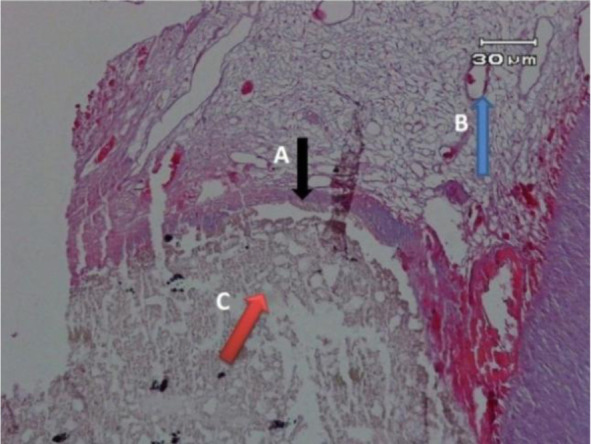
Pathologic image of a sample pulp capped with CEM cement: A (calcified bridge), B (pulp hyperemia), C (CEM material)

**Figure 3:**
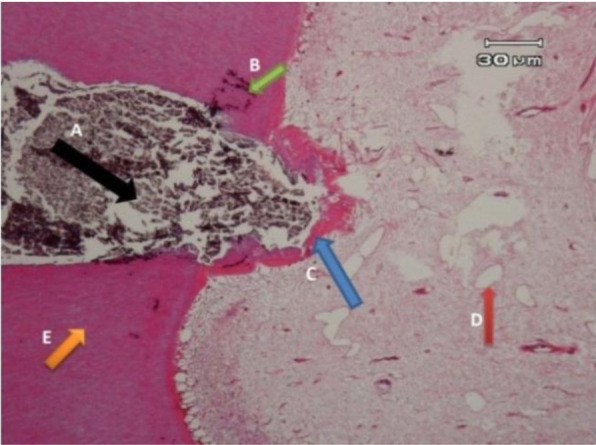
Pathologic image of a sample pulp capped with Biodentine: A (Biodentine material), B (microorganism), C (calcified bridge), D (pulp hyperemia), E (dentin)

Two teeth were excluded; one because of the root fract-ure during the tooth extraction and another one during histological preparation. Finally, 38 teeth were evaluate-d 9 in the MTA group, 9 in the CEM cement group, 10 in the Biodentine group and 10 in the control group. The results of evaluated criteria in different groups are summrized in Table 1. In all samples the pulp was vital. In the control group, mild chronic inflammation was found in 40% of samples. The formation of secondary dentin, similar to the dentin bridge, was found in only two samples (20%). In the MTA group, dentin bridge formation was detected in five samples (55.6%) and none of the specimens showed inflammation (Figure 1). In the CEM cement group, 22.2% of the samples showed mild inflammation and 11.1% showed moderate inflammation. The dentin bridge was formed in 66.7% of the samples (Figure 2). In the Biodentine group, mild and moderate inflammation were found in 60% and 30% of the samples respectively. The dentin bridge for-mation was detected in eight samples (80%) (Figure 3).

**Table1:** The results of evaluated criteria in different groups.

Groups	Pulp vitality	Pulp inflammation				The formation of calcified bridge	The mean thickness of calcified bridge
		Sever	Moderate	Mild	No inflammation		
MTA	100%	100%	-	-	-	55.6%	0.4± 0.419
CEM cement	100%	66.7%	22.2%	11.1%	-	66.7%	0.74 ± 0.79
Biodentine	100%	10%	60%	30%	-	80%	2.85 ± 2.7
Control	100%	60%	40%	-	-	20%	0.24 ± 0.4

The results of statistical analysis showed that the pulpal inflammation observed in the Biodentine group was significantly higher than all groups (*p*= 0.001).

The dentin bridge formation in CEM cement and Biodentine groups were significantly higher than the control group (*p*= 0.045 and *p*= 0.009, respectively), but the difference was not significant between MTA and CEM, and MTA and Biodentine, CEM and Biodentine groups(*p*= 0.63, *p*= 0.26 and *p*= 0.52, respectively). Statistical analysis showed that the mean thickness of dentin bridge was significantly higher in the Biodentine group than that of MTA and control groups (*p*= 0.035, *p*= 0.011 respectively). But there was no significant difference between the other groups (*p*= 0.09).

## Discussion

The present clinical trial study was performed to evaluate the histological response of the human dental pulp to MTA, CEM cement, and Biodentine when used as direct pulp capping materials; the evaluated criteria were more comprehensive than those assessed in previous studies [ 5 , 16 , 19 ]. The main purpose of using the pulp capping materials is to cover pulp and preserve its vitality. In addition, the material’s effect on providing odontoblast-like cells by influencing dental pulp cells is an important factor for improving success rate of DPC [ 20 ]. The results of this study showed that no inflammatory response was observed in the MTA group, whereas Biodentine group showed significantly higher inflammation compared to either MTA, CEM cement or control groups. Tabarsi *et al*. [ 14 ] compared the pulp response during pulpotomy with MTA, CEM and calcium hydroxide in the teeth of dog, and showed that MTA and CEM were significantly better than calcium hydroxide in term of lack of inflammation, but there was no significant difference between CEM and MTA. Faraco and Holland [ 21 ] also reported no inflammation following DPC with MTA, which is consistent with the results of the present study.

One of the most important goals in pulp cap treatment is the reduction or absence of inflammation in the pulp. Less pulp inflammation may indicate the better biocompatibility of pulp-capping materials. [ 22 ] Therefore, induction of less inflammation can be considered as a success factor for pulp-capping materials, whereas dentin bridge formation alone does not prove the healthy status of the pulp. With these interpretations, the MTA and CEM cement showed better results than Biodentine in the terms of inflammatory pulp response.

The results of the present study showed that the pulp necrosis was not observed, which were in agreement with the observations of Katge and Patil [ 5 ] and Nowicka *et al*. [ 16 ]. In the study of Katge and Patil [ 5 ] on decayed human molar teeth, the direct pulp capping was performed with MTA and Biodentine and no necrosis was observed after 6 and 12 months of follow up. Nowicka *et al*. [ 16 ] evaluated the direct pulp capping with calcium hydroxide, Biodentine, MTA, and bonding system on the third human molar, and observed no necrosis in any of the teeth, although the pulp necrosis was seen after pulp capping with MTA in an animal study [ 23 ]. In this study, selected pulp vitality tests were used during the study period. The absence of pulp necrosis in the histological observation justifies the normal response of the tooth to periodic pulp vitality tests, and may indicate that the pulp vitality can be maintained with the presence of the healthy pulp at the start of treatment as well as the use of appropriate technique when working with each one of these capping materials. Therefore, it seems that the type of pulp capping material in maintaining the pulp vitality is less imperative. The results of this study showed that the highest percentage of samples in which the dentin bridge was formed was in the Biodentine group, but there was no significant difference in dentin bridge formation among Biodentine, CEM cement and MTA. This finding is similar to the findings by Katge and Patil [ 5 ], who found no significant difference in the dentin bridge formation between Biodentine and MTA within the six and twelve months. Nowicka *et al*. [ 16 ] reported that the highest dentin bridge formation was related to the Biodentine after pulp capping of the human third molar with calcium hydroxide, MTA, Biodentine and bonding system, but found no significant difference between the MTA and Biodentine in term of dentin bridge formation; which is similar to the result observed in this study. Madani *et al*. [ 19 ] also found no significant difference between MTA and CEM cement in term of dentin bridge formation, in an animal study. 

The results of this study showed that the mean thickness of the dentin bridges produced in the Biodentine group was significantly higher than MTA and control groups, but there was no significant difference between CEM and MTA. Zarrabi *et al*. [ 15 ] compared the pulp responses after pulp capping of human premolar teeth using MTA and the CEM cement, and their results suggested that CEM cement compared to the MTA showed a thicker dentin bridge, but their difference was not significant, which is consistent with the results of the present study. In the study of Novick *et al*. [ 16 ], the maximum thickness of the dentin bridge was produced by Biodentine, followed by MTA, calcium hydroxide and bonding. They showed that the calcium silicate-based materials (Biodentine and MTA) have higher efficiencies in tissue repair than calcium hydroxide, which is probably due to the better stimulation of pulp stem cells by these materials. Tziafa *et al*. [ 17 ] also reported in an animal study that the dentin bridge thickness produced in Biodentine after three and eight weeks was significantly higher than the MTA, which is consistent with the findings of the present study. 

In an animal study, researchers have shown that when comparing direct pulp capping with the Biodentine and MTA, the former material produces more pulp cell stimulation than MTA, which makes thickening of the dentin bridge and the highest amount of ectopic pulp calcification in developing teeth [ 17 , 24 ]. In this study, it should be noted that the pulp capping materials were placed on artificially exposed healthy pulp, and all conditions were ideal and controlled. The results of previous studies showed that the mechanical exposure of the pulp has a much more favorable prognosis and a higher success rate than caries exposure [ 4 , 10 ]. For this purpose, it is suggested that subsequent studies should be performed similar to actual clinical situations and in teeth with deep caries.

## Conclusion

Although the dentin bridge formation and the thickness of the dentin bridge formed in the Biodentine group were higher than the other groups, the amount of pulp inflammation was also higher in contact with this substance. Since one of the main goals of vital pulp therapy is to reduce pulp inflammation and formation of the dentin bridge is not regarded as the success criterion per se, the results of this study suggest that MTA and CEM cement perform better when employed as the direct pulp capping material.
